# Parathyroid Imaging: Past, Present, and Future

**DOI:** 10.3389/fendo.2021.760419

**Published:** 2022-02-25

**Authors:** Michael A. Morris, Babak Saboury, Mark Ahlman, Ashkan A. Malayeri, Elizabeth C. Jones, Clara C. Chen, Corina Millo

**Affiliations:** National Institutes of Health (NIH) Clinical Center, Department of Radiology and Imaging Sciences, Bethesda, MD, United States

**Keywords:** hyperparathyroidism, parathyroid imaging, parathyroid adenoma, parathyroidectomy, scintigraphy, PET, 4D CT MR, oncoradiology

## Abstract

The goal of parathyroid imaging is to identify all sources of excess parathyroid hormone secretion pre-operatively. A variety of imaging approaches have been evaluated and utilized over the years for this purpose. Ultrasound relies solely on structural features and is without radiation, however is limited to superficial evaluation. 4DCT and 4DMRI provide enhancement characteristics in addition to structural features and dynamic enhancement has been investigated as a way to better distinguish parathyroid from adjacent structures. It is important to recognize that 4DCT provides valuable information however results in much higher radiation dose to the thyroid gland than the other available examinations, and therefore the optimal number of phases is an area of controversy. Single-photon scintigraphy with 99mTc-Sestamibi, or dual tracer 99mTc-pertechnetate and 99mTc-sestamibi with or without SPECT or SPECT/CT is part of the standard of care in many centers with availability and expertise in nuclear medicine. This molecular imaging approach detects cellular physiology such as mitochondria content found in parathyroid adenomas. Combining structural imaging such as CT or MRI with molecular imaging in a hybrid approach allows the ability to obtain robust structural and functional information in one examination. Hybrid PET/CT is widely available and provides improved imaging and quantification over SPECT or SPECT/CT. Emerging PET imaging techniques, such as 18F-Fluorocholine, have the exciting potential to reinvent parathyroid imaging. PET/MRI may be particularly well suited to parathyroid imaging, where available, because of the ability to perform dynamic contrast-enhanced imaging and co-registered 18F-Fluorocholine PET imaging simultaneously with low radiation dose to the thyroid. A targeted agent specific for a parathyroid tissue biomarker remains to be identified.

## Purpose of Parathyroid Imaging:* In search of that “reddish brown lymph node”!*

The sole reason for parathyroid imaging is surgical planning, and not diagnosis or risk stratification . This paradigm is different from many others in diagnostic imaging. The diagnosis of primary hyperparathyroidism (HP) and its classification is fundamentally based on plasma biochemical profile not imaging, while the only definitive treatment is surgery which depends on accurate localization. The goal of parathyroid imaging is to localize all sources of excess parathyroid hormone (PTH) secretion pre-operatively.

The history of parathyroid disease and treatment emphasizes the importance of localization. The parathyroid gland was discovered as an anatomic entity in humans less than 150 years ago. Its function was completely unknown for many years. When Von Recklinghausen described a case of a bone abnormality termed ‘osteitis fibrosa cystica’ in 1891, he was not aware of the cause^1^. What he described is what we now know as a brown tumor or ‘Von Recklinghausen disease of bone’. In his autopsy note for this case, he mentioned *“at the left side of the neck below the thyroid gland a reddish brown lymph node is found”* prior to any known connection between these two entities (1).

Twelve years later, Askanazy (2) reported a parathyroid tumor in a case of osteitis fibrosa cystica (1903). The next twenty years were the time of growth in knowledge and confusion at the same time. Similar cases were subsequently reported by Schmorl (3) in 1907, Molineus and Poltauf (4, 5) in 1913, Harbitz (6) in 1915, and Maresch (7) and Schlangenhaufer (8) in 1916. However, considerable controversy was present regarding whether the parathyroid tumor in a series of such cases was the primary (*cause*) or secondary (*effect*) in relationship to the skeletal changes. Erdheim supported a compensatory phenomenon that led him to advocate for supplemental PTH as therapy (9). Schlagenhaufer objected that it would be unusual for only one gland to be involved if the parathyroid enlargement was compensatory, suggesting in 1915 that surgical removal could have a beneficial effect on the bone disease (10). Nevertheless, for some years Erdheim’s views prevailed and Mandl reported treating a patient with animal derived PTH extract and with four human parathyroid glands implantation into the patient without success (11). On July 30th 1925, Mandl removed a parathyroid adenoma measuring 2.5 x 1.5 x 1.2 cm from a patient with bone pain (12), after which the symptoms resolved and the patient remained without recurrence up to 31 years later. This represented the first documented cure of osteitis fibrosa cystica by removal of a parathyroid adenoma.

As the treatment paradigm shifted and resection of parathyroid adenoma became the treatment of choice for primary hyperparathyroidism, the real challenge in the management of hyperparathyroidism presented itself: How are these culprit lesions best localized ?

The importance of accurate localization is illustrated in the case of Captain Martell. He underwent 5 unsuccessful parathyroid surgeries between 1926 to 1930. Finally, the sixth operation was successful, and a parathyroid adenoma was removed from the mediastinum (13–15). This patient illustrates the importance of pre-surgical localization.

Fortunately, significant advances in medicine have been made since Martell’s time. Not only have surgical techniques improved greatly, multiple new imaging modalities are now available that can localize parathyroid adenomas preoperatively and even intraoperatively. While no single method is perfect, the vast majority of parathyroid adenomas are now localized preoperatively through a variety of imaging approaches, often using a combination of techniques such that successful resection can be achieved with minimal harm to the patient. Ultrasound is most applicable for evaluating the superficial acoustic field of the neck. Various radiopharmaceuticals have been utilized in single photon planar scintigraphy or single photon compute tomography (SPECT) imaging and more recently positron emission tomography (PET) imaging, however none target a specific biomarker of parathyroid pathology at this time. CT and MR rely on hemodynamic and vascular features but these are also non-specific with variable reliability. More invasive techniques include angiography and biopsy.

## Overview of Parathyroid Characteristics: Essentials for Imaging

Increased PTH secretion is the definition of HP. Primary HP is diagnosed when the PTH level is elevated inappropriately compared to the level of serum calcium as the result of autonomous overproduction by parathyroid tissue. The etiology is most commonly a parathyroid adenoma (PA) (85%), followed by multiple adenomas (15-20%), parathyroid hyperplasia (<15%), and parathyroid carcinoma (~1%) (16). Increased PTH secretion is an important cause of hypercalcemia with an incidence of up to 5 cases out of 1000 adults. The disease is more common in women and above age 50 years old. HP may be associated with radiation exposure, sarcoidosis, and a number of familial disorders such as MEN1. There is loss of heterozygosity on chromosome 11 (where the MEN1 gene is located) in 25-40% of cases of primary hyperparathyroidism (17). Normocalcemic HP can also occur and rarely presents with osseous disease (18).

### Evolutionary Biology and Embryology

PTH is present in chordates and becomes important evolutionarily with the development of cartilaginous and bony structures. In vertebrates, up to six PTH and three PTH receptor (PTHR) paralogs have been identified, varying in number between mammals and teleost fish. At least two species of fish express PTH, however its definitive source and physiological function earlier in evolutionary phylogeny remains to be determined (19).

In humans, there are typically 4 paired parathyroid glands, two superior and two inferior. However, 10% of people have 2-3 glands, 5% of people have 5 glands, and 0.2% of people have 6 glands and the number of supernumerary glands in humans has ranged up to 11 in an autopsy series (20). During normal human development, the superior parathyroid glands originate from the fourth pharyngeal pouch, descending with the thyroid gland and eventually coming to rest near the cricothyroid junction, posterior to the thyroid and recurrent laryngeal nerve. Inferior parathyroid glands originate from the third pharyngeal pouch, descending with the thymus and are usually found near the inferior thyroid, anterior to the recurrent laryngeal nerve. The parathyroid glands can be found anywhere along the pathway of descent of the branchial pouches, hence their notoriously variable location.

### Anatomy and Histology

Each normal parathyroid gland is a yellow-brown organ of approximately 25-40 mg. A weight greater than 60 mg is considered abnormally large. Glands are composed primarily of chief cells with fat and thin fibrous capsules dividing the gland into lobules. There are also Oxyphil cells which are larger than the chief cells that have acidophilic cytoplasm due to mitochondria with no secretory granules. Stromal fat is also present. The percent fat content is related to constitutional fat content with a mean stromal fat around 17% (21). The parathyroid stains positive for chromogranin A, PTH, GATA3, parafibromin (CDC73), and synaptophysin.

Parathyroid tissue may have a pseudofollicular pattern resembling thyroid follicles, however it is differentiated from thyroid tissue in that it lacks birefringent calcium oxalate crystals detectable by polarized light microscopy (22).

#### Ectopic Parathyroid

Ectopic parathyroid tissue has an incidence as high as 35% due to aberrations in migration during the early stages of development. Like entopic parathyroid tissue, it can be hyperplastic, symptomatic, and associated with secondary HP. It is a common cause of persistent or recurrent HP and failure of parathyroid surgery (23, 24). It can present as symmetrical even when ectopic (25). Ectopic parathyroid thyroid tissue can become adenomatous and cause primary HP, hypercalcemia, and other associated symptomatology (23).

Ectopic parathyroid glands occur along the path of migration of the branchial pouches from the level of the carotid sheath to the heart. Ectopic superior parathyroid glands are most frequently located posteriorly near the tracheoesophageal groove or retroesophageal region (24). Ectopic locations of the inferior parathyroid glands most typically occur in the anterior mediastinum in association with the thymus gland or thyroid gland (26). Ectopic parathyroid glands can also occur along the course of the vagus nerves (27), within the thyroid gland (28), pyriform sinus (29), retropharyngeal region, and axilla (30).

#### Intrathyroid Parathyroid

A parathyroid gland, whether normal or abnormal, surrounded entirely by thyroid parenchyma with no capsule is considered an intrathyroidal parathyroid and occurs due to aberrant migration of the parathyroid glands during embryogenesis (31). Intrathyroidal parathyroid can be confused with intracapsular parathyroid gland, which is a parathyroid gland situated within the crevices of the thyroid (32, 33).

Intrathyroidal parathyroid glands are rare but intrathyroidal parathyroid tissue is not. In a survey of 350 children, the presence of parathyroid and thymic tissue within the thyroid gland was suggested to be so common that it is a normal occurrence (34). Intrathyroidal parathyroid tissue was found in 3% of infants on routine sections and 70% on step sections (34). Intrathyroidal parathyroid glands may develop adenomatous and hyperplastic pathologies like other parathyroid glands (35, 36).

The incidence of true functioning intrathyroidal parathyroid gland represents less than 1% of all hyperparathyroidism cases in a large series (37, 38) with 3:1 female predominance. Over 400 cases of intrathyroidal parathyroid adenoma have been described in case reports and series, with less than 10 cases of intrathyroidal parathyroid carcinoma identified. Intrathyroidal parathyroid is three times more likely to be in the superior pole of the thyroid and slightly favors the right over the left.

### Physiology and Molecular Biology

PTH is a peptide hormone consisting of 84 amino acids. It is derived from pre- pro- PTH and its release is related to the ionized calcium level and its own negative feedback. The biological activity results from 34 amino acid residues at the amino terminus. Other portions are inert but can result in false positives in detection systems. The PTH receptor is a G-protein coupled receptor resulting in the transduction of the cAMP pathway and the production of phosphatidylinositol diphosphate. PTH related protein (PTHrP) is rarely produced by benign lesions (39).

### Pathology: Non-Neoplastic

#### Parathyroid Hyperplasia

In parathyroid hyperplasia there is typically more than one gland involved and the weight of all of the hyperplastic glands usually measures between 1-3 g. Hyperplastic parathyroid cells may show clonality and involve mainly the chief cells. Parathyroid hyperplasia can be sporadic or in patients with a history of prior radiation to the neck. In the setting of MEN1 or 2A patients may begin with multigland hyperplasia and progress to multiple adenomas. Hyperplasia of parathyroid adipose tissue is rare.

In primary chief cell hyperplasia, there is increased production of PTH associated with MEN1 or MEN2A. There is no association with MEN2B. Parathyromatosis, when microscopic foci of hyperplastic parathyroid tissue are found in the neck, is associated with chief cell hyperplasia and prior surgery (40). Bilateral primary chief cell hyperplasia is associated with loss of the APC gene (41).

#### Parathyroid Cyst

Cystic parathyroid lesions often contain turbid or colored fluid, present at all ages, and can be diagnosed by FNA where high PTH is found in the fluid (42). Cysts measure 1-10 cm and are unilocular, and thin walled.

Parathyroid cysts are a rare cause of neck swelling, accounting for 0.6% of thyroid and parathyroid lesions (43). Functional parathyroid cysts are more common than nonfunctional parathyroid cysts (44). Patients are usually normocalcemic and present with an asymptomatic neck mass, including in the low cervical region and anterosuperior mediastinum. They may occur in a hyperplastic gland (45), due to cystic degeneration within an adenoma, or within heterotopic salivary gland-like tissue (46).

Patients with large cysts should be managed expectantly to avoid development of symptomatic hypocalcemia. Cyst rupture should be avoided during resection. Many cysts are amenable to aspiration for diagnosis and treatment and ethanol ablation can be considered for recurrent cases (47). Cystic parathyroid lesions are a common cause of false negative results on molecular imaging.

### Pathology: Neoplastic

#### Adenoma

**Figure 1:**
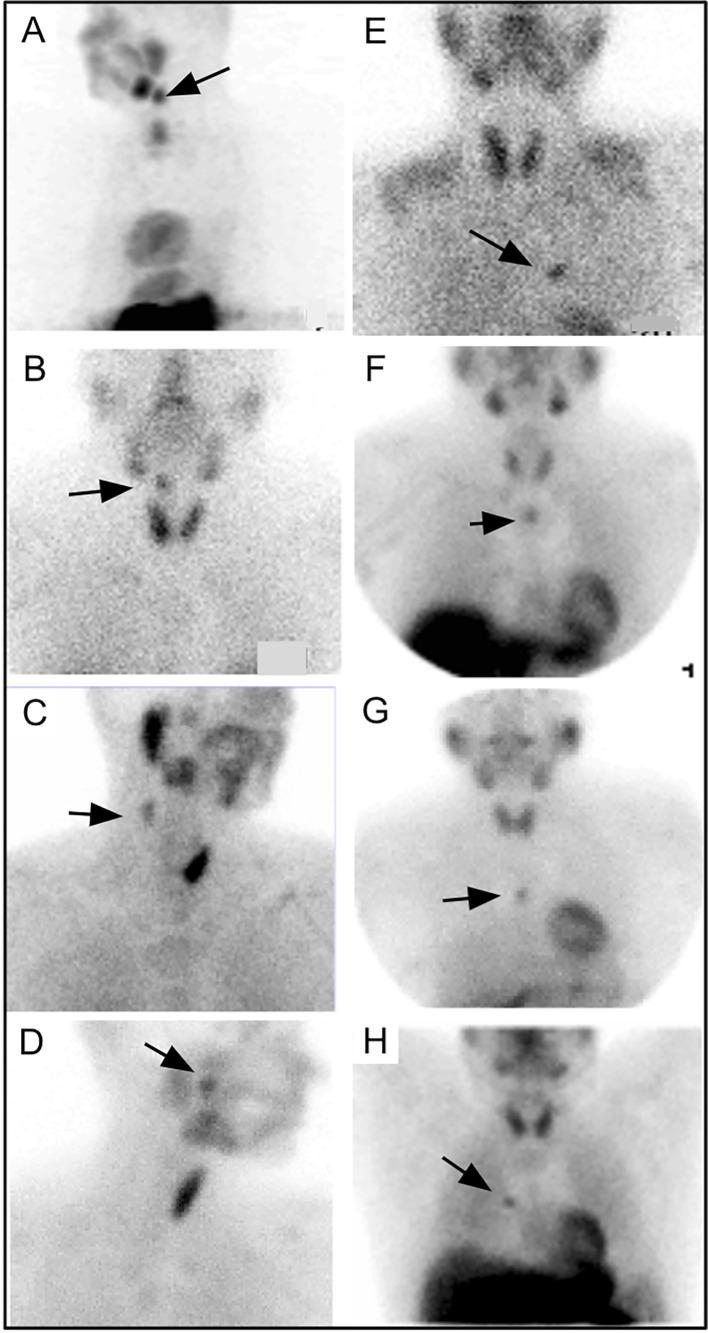
Examples of ectopic PA (arrows) occurring in the neck **(A–D)** and mediastinum **(E–H)**.

Parathyroid adenoma (PA) typically involves a single gland and represents neoplasia of parathyroid cells with nuclear pleomorphism more common than in hyperplasia (48). Chief cells within parathyroid adenomas are most commonly responsible for elevated PTH levels (49–51). Parathyroid adenomas more commonly involve the inferior glands over superior glands. The diagnosis of parathyroid adenoma is typically derived from an intraoperative finding of solitary, or less commonly two, enlarged parathyroid gland(s) with histology of a hypercellular parathyroid tumor and compressed adjacent normal parathyroid tissue. Adenomas can occur in ectopic locations similar to normal parathyroid tissue, which are found in up to 16% of cases and most commonly occur in the neck or mediastinum (**Figure 1**) (20, 52, 53). Simultaneous adenomas can occur involving multiple glands (54). Parathyroid adenoma can mimic follicular thyroid neoplasm at FNA.

The incidence of parathyroid adenoma has increased over the past 50 years, perhaps due to routine biochemical testing, with adenomas often detected early in asymptomatic patients. Most are sporadic cases of unknown etiology. Women are more frequently affected and it is common for adenomas to occur in the 30s-60s (55). When symptoms occur in the setting of PA, they are typically related to hypercalcemia due to primary HP.

Syndromes associated with parathyroid adenoma include hyperparathyroidism jaw tumor syndrome (HRPT2 gene germline mutation) and multiple endocrine neoplasia (MEN1 > MEN2A) (54). Risk factors include radiation exposure and long-term lithium therapy (56, 57).

#### Parathyroid Carcinoma

Parathyroid carcinoma is rare. At a large tertiary cancer center within the United States, a study identified only 20 patients over a 15-year period who had been operated on for parathyroid carcinoma with at least one preoperative imaging exam at the same institution (58). Conventional structural imaging may be helpful to identify this entity as it is typically poorly circumscribed, which can distinguish it from parathyroid adenoma (59). The mean diameter of parathyroid carcinomas at histopathology is suggested to be 3.4 cm with a weight of 19.2 g (60), considerably larger than the typical adenoma. The role of imaging in parathyroid carcinoma is primarily for evaluation of metastatic disease for which FDG PET/CT has demonstrated promise (61, 62).

#### Secondary Neoplasia

Rarely, neoplasms can involve the parathyroid through metastasis or direct involvement. A literature review of 127 reported cases revealed the most common primary malignancies were breast carcinoma (66.9%), melanoma (11.8%), and lung carcinoma (5.5%) (63). Patients with metastatic parathyroid lesions typically had widespread metastatic disease and were reported to develop abnormal calcium homeostasis even greater than those with primary parathyroid disease. Thyroid neoplastic disease is reported to typically secondarily involve the parathyroid gland by direct extension (64). These entities are potential masqueraders of parathyroid disease on imaging.

### Parathyroid Imaging in Current Clinical Practice

Management for patients with hyperparathyroidism is complex and multi-disciplinary. While the diagnosis is made by internal medicine physicians (such as endocrinologists), the curative treatment is performed by surgeons. Without a positive finding on imaging, it is unlikely that clinicians refer patients for definitive surgery (65). Parathyroid imaging is cost saving. For instance, a study from Frank et al. in 2020 found that whereas bilateral neck exploration costs $9578 with a success rate of 97.3%, single photon emission computed tomography (SPECT) imaging and minimally invasive parathyroidectomy (MIP) cost $8197 with a success rate of 98.6% (66). Contrary to some claims undermining the utility of preoperative imaging, the American Head and Neck Society (AHNS) Endocrine Section emphasized the necessity of precision preoperative localization to avoid unnecessary repeat operation (67, 68) and to address the concurrent thyroid pathology, which can be identified on parathyroid imaging, in the same operation (69, 70).

#### Molecular Parathyroid Imaging

**Figure 2:**
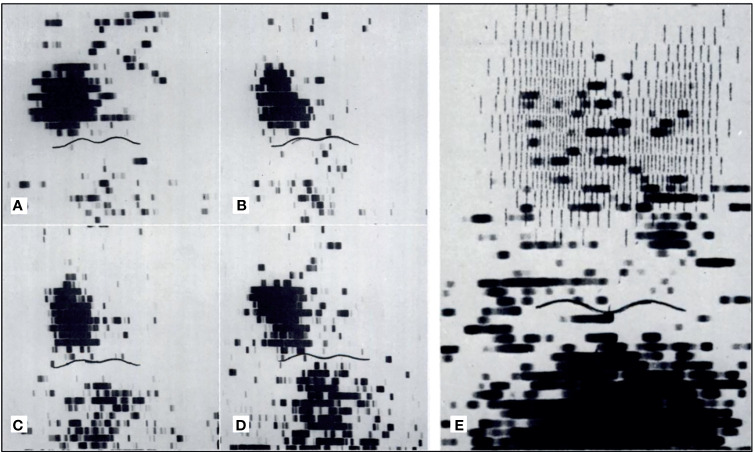
Early parathyroid scintigraphy using ^75^Se depicting a 2.3g parathyroid adenoma on the right. **(A, B)** 10 and 45 minutes after intravenous administration of radiotracer with tumor tracer localization and low tracer activity in the sternal region. **(C, D)** 2 h and 24 h after radiotracer administration with tumor tracer localization and increasing radiotracer activity in the sternal region. **(E)** Superimposition of thyroid scan onto parathyroid scintigraphy with 2.7 g parathyroid adenoma inferior to the left lobe of the thyroid. Reproduced with permission from: *Colella AC, Pigorini F. Experience with parathyroid scintigraphy. Am J Roentgenol Radium Ther Nucl Med. 1970 Aug;109(4):714-23. doi: 10.2214/ajr.109.4.714. PMID: 5451873*.

**Figure 3:**
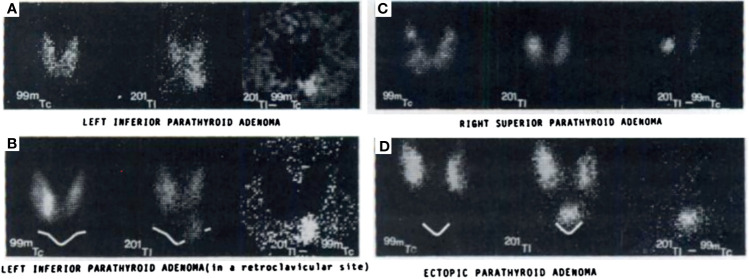
Early examples of parathyroid pathology detection using ^99m^Tc-pertechnetate and thallium subtraction scintigraphy. Upper left panel **(A)** depicts ^99m^Tc-pertechnetate thyroid uptake on the left image, ^201^Tl uptake in the middle image, and the subtraction image highlighting a left inferior parathyroid adenoma on the right image. Lower left panel **(B)** depicts a similar ^201^Tl minus ^99m^Tc-pertechnetate series of three images of a left inferior parathyroid adenoma with decreased signal to noise ratio due to its location behind the clavicle contributing to increased attenuation relative to the series above. Right upper panel **(C)** depicts a similar subtraction series of a right superior parathyroid adenoma. Right lower panel **(D)** depicts a subtraction series of an ectopic parathyroid adenoma near the sternal notch. Reproduced with permission from: *Ferlin G, Borsato N, Camerani M, Conte N, Zotti D. New perspectives in localizing enlarged parathyroids by technetium-thallium subtraction scan. J Nucl Med. 1983 May;24(5):438-41. PMID: 6842292*.

In the 1970s, imaging of the parathyroid gland was initially attempted with ^75^Se methionine (71). Further advancement through double-tracer image subtraction techniques remained suboptimal [**Figure 2** (71)]. The intention of these subtractions was to separate thyroid lesions from parathyroid lesions using the second radiotracer [^125^I (72), or ^131^I (73), or ^99m^Tc-pertechnetate (74)] in addition to ^75^Se-methionine. ^201^Tl was then investigated for parathyroid imaging with ^99m^Tc-pertechnetate subtraction, which in the 1980s became the standard of care (75–78) [**Figure 3** (75)]. However, ^201^Tl was suboptimal for imaging due to its physical characteristics.

##### From Double-Tracer Paradigm to Single-Tracer Dual-Phase Paradigm

During the 1980s, an effort to find a better cardiac perfusion radiotracer to overcome limitations of ^201^Tl lead to development of many tracers. After the first few failures, ^99m^Tc-TBIN (tertiary butyl isonitrile) was the first radiotracer with some clinical promise (79). The cationic isonitrile complex eventually lost favor due to its suboptimal biodistribution, with particularly high pulmonary and hepatic uptake. Subsequent endeavors lead to the development of analog ^99m^Tc-sestamibi (sesta-methoxy-isobutyl-isonitrile), which was FDA approved for cardiac imaging in 1990 (80–82). ^99m^Tc-sestamibi also demonstrated uptake in the glands of the neck. In 1989, Coakley and colleagues observed a differential washout rate of ^99m^Tc-sestamibi from the parathyroid relative to thyroid (83, 84). This led to the development of dual phase imaging for parathyroid disease, with identification of parathyroid lesions based on their delayed washout compared to thyroid tissue (85). It is important to note ^99m^Tc-tetrofosmin does not have the same differential kinetics and is not interchangeable in dual-phase single agent parathyroid imaging with ^99m^Tc-sestamibi (86).

**Figure 4:**
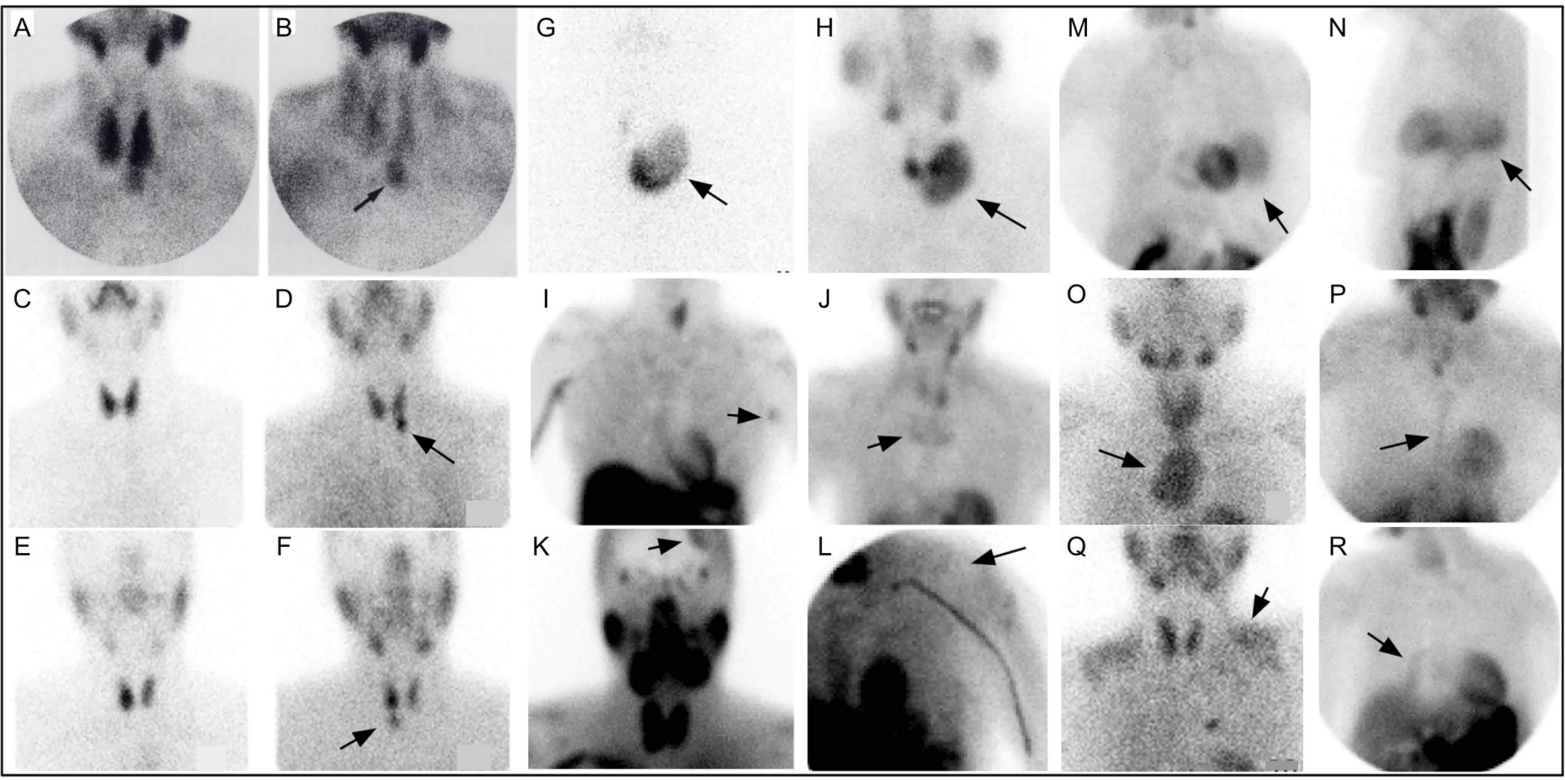
Early ^99m^Tc-sestamibi parathyroid imaging **(A)** with uniform uptake within the thyroid and more mild focal uptake in the left lower neck, inferior to the thyroid. **(B)** Delayed image shows decreased radiotracer uptake within the thyroid and increased radiotracer uptake within a 2.5g parathyroid adenoma (arrow). Example of dual tracer method **(C–F)** with left image depicting 99mTc-pertechnetate and right image depicting 99mTc-sestamibi showing a left PA **(D)** and right PA **(F)**. Uptake not related to PA (arrows) can also be seen **(G–R)**. Multinodular goiter 99mTc-pertechnetate **(G)** and 99mTc-sestamibi **(H)**. Also depicted on 99mTc-sestamibi are **(I)** left axillary skin fold, **(J)** manubrium brown tumor, **(K)** meningioma, **(L)** deltoid implant, **(M)** diaphragmatic hernia anterior and **(N)** lateral, **(O)** retropharyngeal PA and goiter extending into mediastinum, **(P)** sternotomy, **(Q)** muscle uptake, **(R)** atelectasis adjacent to mediastinum on the right. Reproduced with permission from: *Taillefer R, Boucher Y, Potvin C, Lambert R. Detection and localization of parathyroid adenomas in patients with hyperparathyroidism using a single radionuclide imaging procedure with technetium-99m-sestamibi (double-phase study). J Nucl Med. 1992 Oct;33(10):1801-7. PMID: 1328564*.

Following Coakley’s report, ^99m^Tc-sestamibi was utilized to detect parathyroid adenomas (83, 84) [**Figure 4** (85)]. In 1992, a prospective study supported the role of ^99m^Tc-sestamibi for localization of parathyroid adenomas in patients with known primary HP (85).

On a cellular level, sestamibi uptake is postulated to be concentrated in mitochondria-rich oxyphil cells (87), which are present in high numbers in parathyroid adenomas (88). Sestamibi is a substrate of permeability glycoprotein (P-gp), also known as multidrug resistance protein 1 (MDR1), a membrane transporter in the ATP-binding cassette (ABC) family thought to pump various substrates including many toxins, chemotherapy agents, and xenobiotics out of cells (89). It was recently reported that vascular endothelial growth factor type 2 (VEGFR-2) expression was significantly less in ^99m^Tc-sestamibi negative parathyroid adenomas (90). It remains to be determined whether vascular permeability plays a role in sestamibi uptake. Sestamibi does not target a biomarker specific to parathyroid disease.

From the dawn of its usage, Sestamibi was observed to have uptake in numerous pathologies, such as differentiated thyroid cancer (91), breast neoplasia (92, 93), nasopharyngeal carcinoma (94), and lymph nodes in Castleman’s disease (95). It complicates matters that collision tumors involving off target pathology have also been reported to occur synchronously with parathyroid adenomas (96). Cystic adenomas have been reported to lack sestamibi uptake (97).

If possible, patients should avoid vitamin D or calcimimetics for two weeks prior to parathyroid imaging with sestamibi as it is reported that these can decrease localization (98). Early data suggests calcium channel blockers could decrease uptake of ^99m^Tc-sestamibi with the odds ratio of a negative scan 2.88 in patients taking calcium channel blockers (99). More data is needed to better elaborate recommendations related to the use of these agents during ^99m^Tc-sestamibi scanning.

##### Combining Double-Tracer and Dual-Phase Strategies: A Necessity to Address Thyroid Nodules

After the introduction of dual phase imaging, it became apparent that the combined sensitivity of dual phase with subtraction imaging was higher than either technique alone. Chen et al. reported dual-tracer studies using both ^123^I or ^99m^Tc-pertechnetate plus ^99m^Tc-sestamibi by visual inspection provided superior detection of parathyroid adenoma compared to single tracer dual phase and that computer subtraction may not be necessary (100, 101). Indeed, Chen also pointed out that parathyroid adenoma can concentrate ^99m^Tc-pertechnetate in some cases, potentially confounding subtraction images (102).

**Figure 5:**
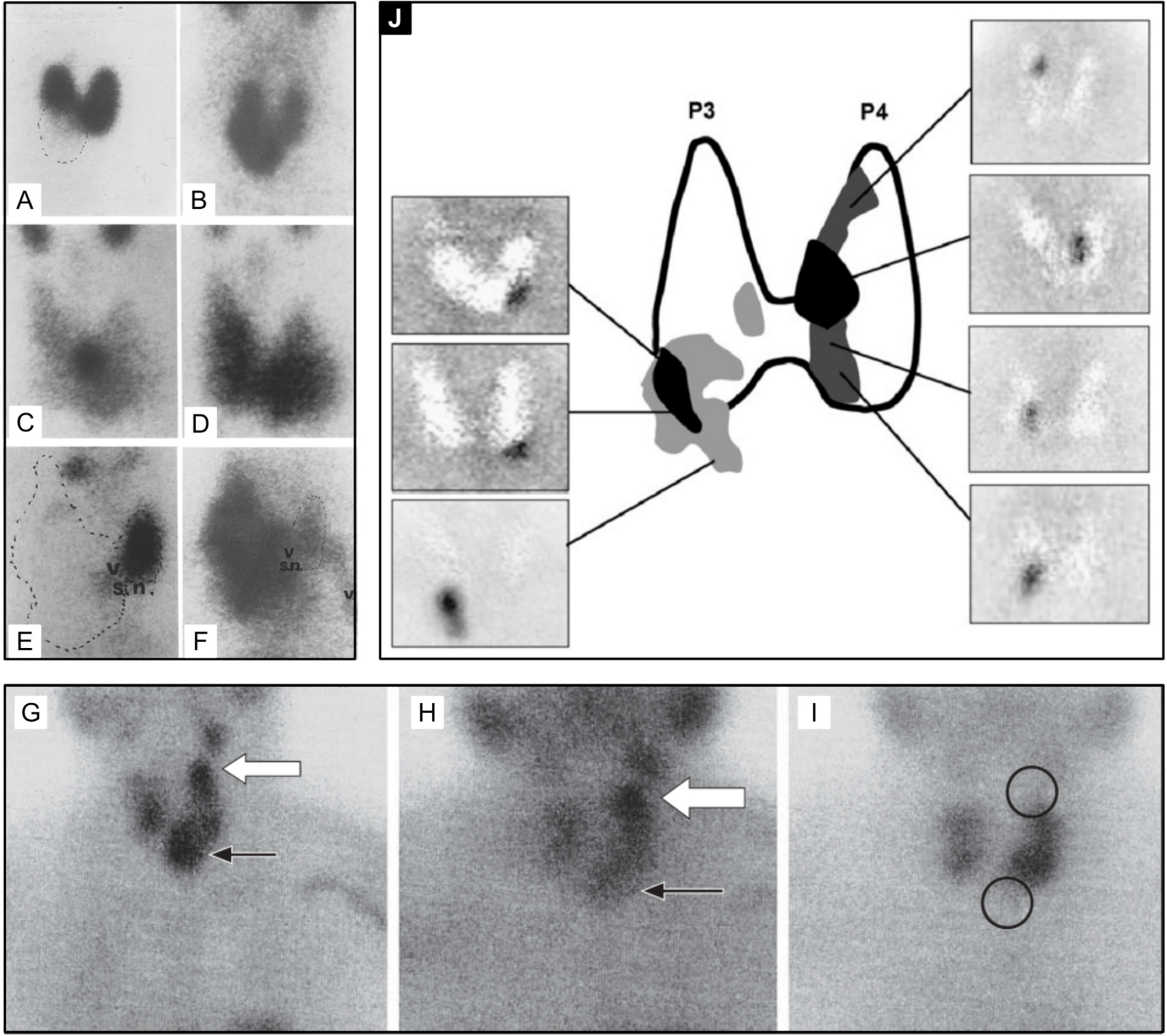
99mTc-sestamibi with or without 99mTc-pertechnetate imaging is the current standard of care for PA detection at most institutions at the time of writing of this article. Thyroid nodules may confound parathyroid imaging using this method as they have variable uptake relative to the thyroid gland with 99mTc-pertechnetate **(A, C, E, I)** and with 99mTc-sestamibi **(B, D, F, G, H)**. Although benign thyroid nodules typically washout on delayed 99mTc-sestamibi imaging **(H)**, thyroid carcinoma [**(G, H)**, white arrow] can have retained uptake similar to parathyroid adenoma [**(G, H)**, black arrow]. Panel **(J)** provides an atlas of PA appearances on dual tracer 99mTc-sestamibi minus 99mTc-pertechnetate subtraction and the frequency of PA locations relative to the thyroid gland (highest frequency-black, lower frequency-grey). Superior PA are derived from the fourth pharyngeal pouch (P4) and therefore more posterior. Inferior PA are derived from the third pharyngeal pouch and therefore more anterior (P3). SPECT imaging can aid in distinguishing the anterior versus posterior location. Reproduced with permission from: **(A–F)** Földes I, Lévay A, Stotz G. Comparative scanning of thyroid nodules with technetium-99m pertechnetate and technetium-99m methoxyisobutylisonitrile. Eur J Nucl Med. 1993;20: 330–333. doi: 10.1007/BF00169809. **(G–I)** Lorberboym M, Ezri T, Schachter PP. Preoperative technetium Tc 99m sestamibi SPECT imaging in the management of primary hyperparathyroidism in patients with concomitant multinodular goiter. Arch Surg. 2005;140: 656–660. doi: 10.1001/archsurg.140.7.656. **(J)** Taïeb D, Hassad R, Sebag F, Colavolpe C, Guedj E, Hindié E, et al. Tomoscintigraphy improves the determination of the embryologic origin of parathyroid adenomas, especially in apparently inferior glands: imaging features and surgical implications. J Nucl Med Technol. 2007;35: 135–139. doi: 10.2967/jnmt.107.039743.

Although differential washout is useful for differentiation of normal thyroid and parathyroid pathology, solid thyroid nodules have a low washout rate, similar to parathyroid. This ^99m^Tc-sestamibi retention phenotype is independent of the functionality (hot or cold on ^99m^Tc-pertechnetate scan) and independent of benignity *versus* malignancy (103). Association of nodular goiter with hyperparathyroidism (104, 105) makes the washout similarity of thyroid nodules and parathyroid adenoma a pragmatic conundrum [**Figure 5** (103, 105, 106)].

##### Current First Line Molecular Imaging Approach

Multiple studies in patients undergoing repeat operations, including a cohort of 237 patients at the National Institutes of Health, found dual tracer multi-phase ^99m^Tc-pertechnetate and ^99m^Tc-sestamibi planar nuclear imaging in combination with delayed SPECT and ultrasound to provided the highest accuracy of the non-invasive approaches at the time (107, 108). More advanced techniques not widely available at the time, such as PET imaging, were not evaluated.

**Figure 6:**
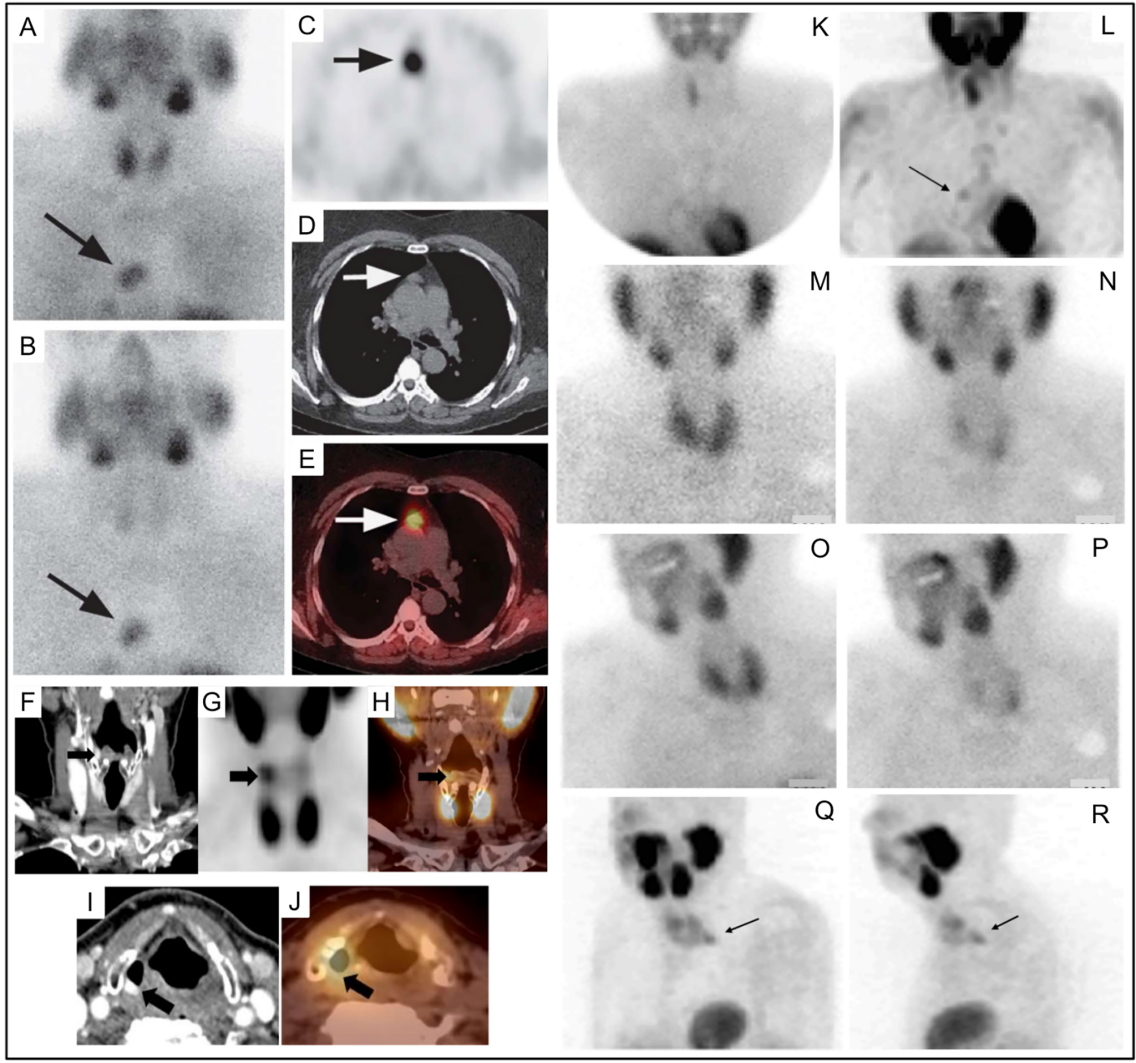
Parathyroid planar imaging should include the mediastinum, where a large percentage of ectopic PA occur. Planar scintigraphy of an ectopic PA **(A)** 20min and **(B)** delayed 2h. ^99m^Tc-sestamibi SPECT/CT imaging can aid in anatomical localization and is particularly helpful for ectopic PA. ^99m^Tc-sestamibi SPECT/CT of mediastinal PA on axial SPECT [**(C)**-black arrow], axial CT [**(D)**-white arrow], and axial fused SPECT/CT [**(E)**-white arrow]. ^99m^Tc-sestamibi SPECT/CT of an ectopic PA in a particularly rare position near the right piriform sinus on coronal CECT [**(F)**-black arrow], coronal SPECT [**(G)**-black arrow], coronal fused SPECT/CT [**(H)**-black arrow], axial CECT [**(I)**-black arrow], axial fused SPECT/CT [**(J)**-black arrow]. SPECT imaging can also be helpful when planar imaging is negative **(K, M, N)** as in **(L)** showing mediastinal PA (arrow) adjacent to the cardiac border. Another PA behind the left lobe of the thyroid was not identified on planar anterior early 99mTc-sestamibi **(M)**, delayed 99mTc-sestamibi **(N)**, or on planar oblique images **(O, P)**, but was identified on SPECT [**(Q, R)**, arrows]. Reproduced with permission from: **(A–E)** *Wong KK, Fig LM, Gross MD, Dwamena BA. Parathyroid adenoma localization with 99mTc-sestamibi SPECT/CT: a meta-analysis. Nucl Med Commun. 2015 Apr;36(4):363-75. doi: 10.1097/MNM.0000000000000262. PMID: 25642803.* **(F–J)** *Hsieh MP, Nemer JS, Beylergil V, Yeh R. Ectopic Parathyroid Adenoma of the Piriform Sinus on Parathyroid 4D-CT and 99mTc-MIBI SPECT/CT. Clin Nucl Med. 2020 Aug;45(8):e358-e359. doi: 10.1097/RLU.0000000000003163. PMID: 32558723*.

Today, the majority of parathyroid scintigraphy is performed in conjunction with SPECT or SPECT/CT where available. A systematic review of 24 studies using ^99m^Tc-sestamibi SPECT/CT in 1276 patients between January 2003 and March 2014 suggested an estimated sensitivity of 86% on a per patient basis compared with 74% for SPECT and 70% for planar techniques (109). In the same study, SPECT/CT also outperformed SPECT and planar techniques for ectopic parathyroid adenomas which occurred in up to 20% of subjects [**Figure 6** (109, 110)].

#### Ultrasound

High resolution neck ultrasound is an accessible structural imaging technique that is capable of visualizing diseased parathyroid glands without the use of ionizing radiation. The cost of neck ultrasound is approximately $200, with other imaging studies costing over $1000 (68, 111). The examination is best performed with a linear array high frequency probe (7.5-13 MHz) by transverse and longitudinal scanning. The parathyroid ultrasound should include the paratracheal spaces, the carotid-jugular axis with the superior aspect reaching the carotid bifurcation, and thyroid gland with the inferior aspect reaching the sternal notch (112–115).

**Figure 7:**
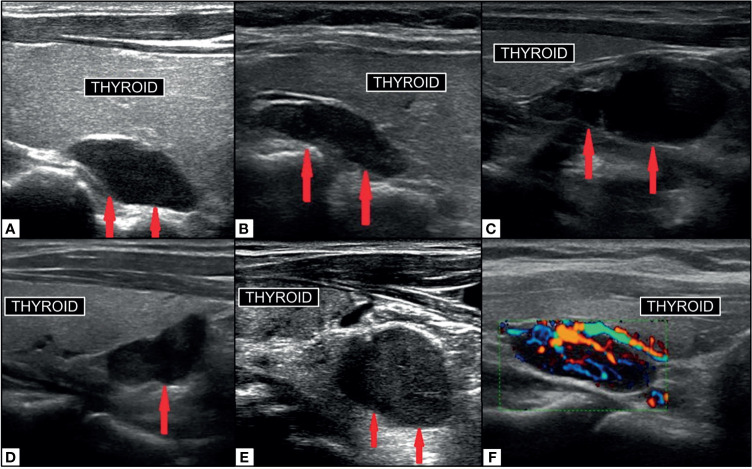
Ultrasound imaging of parathyroid adenoma (red arrows). Right superior PA **(A)** in median retrothyroidal location, hypoechoic, measuring 2.2cm. Left superior PA **(B)** in the upper retrothyroidal location, hypoechoic, measuring 2.3 cm. Predominantly cystic right inferior parathyroid adenoma **(C)** adjacent to lower pole of thyroid, anechoic, measuring 3.5 cm. Right inferior PA **(D)** in the lower retrothyroidal location, hypoechoic, measuring 2 cm. Left inferior PA **(E)** near the lower pole of thyroid extending to the retrosternal region, hypoechoic, measuring 2.3 cm. PA on color-doppler ultrasonography **(F)** with central hyperemia and afferent ‘polar’ vessel. Reproduced with permission from: *Vitetta GM, Ravera A, Mensa G, Fuso L, Neri P, Carriero A, Cirillo S. Actual role of color-doppler high-resolution neck ultrasonography in primary hyperparathyroidism: a clinical review and an observational study with a comparison of 99mTc-sestamibi parathyroid scintigraphy. J Ultrasound. 2019 Sep;22(3):291-308. doi: 10.1007/s40477-018-0332-3. Epub 2018 Oct 24. PMID: 30357759; PMCID: PMC6704209*.

The morphology of a parathyroid adenoma on ultrasound is described as an oblong or ovoid shaped, hypoechoic structure with uniform echogenicity, with echogenic capsule, which is typically hypervascular on color doppler around the capsule and centrally. Internal heterogeneity can result from fat, hemorrhage, or calcification. The parathyroid may have an identifiable feeding vessel at the pole (68) [**Figure 7** (116)]. The normal parathyroid gland is difficult to reliably identify on ultrasound. Asking the patient to swallow can increase conspicuity of the inferior glands. The parathyroid gland should not be confused with lymph nodes which have a feeding vessel leading to the hilum which is typically echogenic due to fat. By now, it is widely recognized that ultrasound can be helpful as a quick, well tolerated, cost effective first line imaging exam for patients with primary HP. Ultrasound is rarely the only preoperative imaging examination performed, and should not be relied upon in isolation in the usual standard of care for these patients.

The AHNS Endocrine Section 2019 guidelines also suggest a benefit of intraoperative ultrasound to more precisely guide the surgical approach and localization (68). They also suggest that in rare instances, PTH assay of an ultrasound guided fine needle aspiration (FNA) of an indeterminate lesion along the posterior aspect of the thyroid can have a benefit in distinguishing intrathyroidal parathyroid from a thyroid nodule. There is however a risk of seeding parathyroid tissue and in most cases FNA of parathyroid lesions is not necessary or recommended. Molecular or structural imaging has been suggested to provide an anatomical map with which to compare intraoperative findings.

#### Dynamic Enhanced Computed Tomography (4DCT)

**Figure 8:**
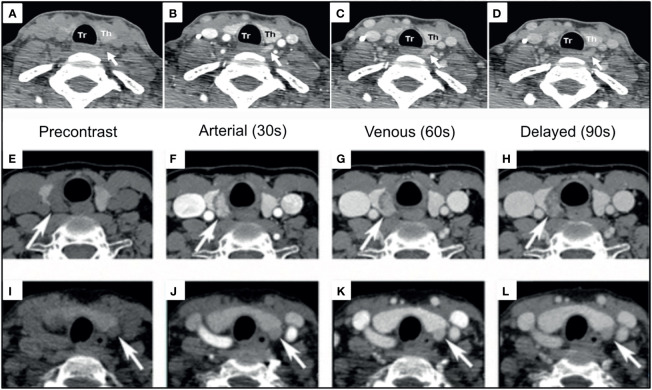
4DCT imaging of parathyroid adenomas. Left PA [**(A–D)**, white arrows] and Right PA [**(E–H)**, white arrows] are visualized as arterially enhancing soft-tissue structures adjacent to the thyroid, which wash out on subsequent phases. Left PA [**(I–L)**, white arrows] with progressive enhancement greatest on the venous phase demonstrating enhancement characteristics can be variable. Reproduced with permission: **(A–D)** *Mahajan A, Starker LF, Ghita M, Udelsman R, Brink JA, Carling T. Parathyroid four-dimensional computed tomography: evaluation of radiation dose exposure during preoperative localization of parathyroid tumors in primary hyperparathyroidism. World J Surg. 2012 Jun;36(6):1335-9. doi: 10.1007/s00268-011-1365-3. PMID: 22146947.* **(E–L)** *Lee EK, Yun TJ, Kim JH, Lee KE, Kim SJ, Won JK, Kang KM, Choi SH, Sohn CH. Effect of tumor volume on the enhancement pattern of parathyroid adenoma on parathyroid four-dimensional CT. Neuroradiology. 2016 May;58(5):495-501. doi: 10.1007/s00234-016-1656-3. Epub 2016 Feb 5. PMID: 26847704*.

To identify the source of PTH excessive secretion, multiphasic dynamic contrast enhanced CT imaging was first implemented in 2006 (117). This technique, termed 4DCT, uses both morphological and enhancement patterns of lesions to identify and differentiate them from mimickers such as lymph nodes or the thyroid gland (68, 118, 119). The typical protocol is either 4 phases or 3 phases: a non-contrast phase, followed by an arterial phase (25-30 seconds after contrast bolus), a venous phase (~30 seconds after arterial phase), with or without delayed venous phase (~60 seconds after the arterial phase) (68, 118) [**Figure 8** (120, 121)].

Benefits of 4DCT include: (1) short imaging time and (2) high spatial resolution to detect small and ectopic glands. Drawbacks include high radiation dose to the thyroid at approximately 92 mGy with 4DCT compared to 1.6 mGy with ^99m^Tc-sestamibi, or about 57 times more radiation (120, 122). Higher radiation dose could, at least in theory, contribute to increased risk of future thyroid malignancy and should be cautioned particularly in younger patients. Although 4DCT sensitivity for single adenomas was reported up to 94% in one study, the sensitivity for multigland disease (MGD)–which is a common presentation—is reported less than 60% (118). Sho et al. reported the sensitivity of 4DCT for MGD is as low as 32-53% (123). It is possible that modality independent factors, such as the field of view or satisfaction of search could contribute in part to these results.

The principle of 4DCT is based on the assumption that PA has distinct enhancement kinetics. In general, lymph nodes progressively enhance and are darker on earlier phases of CT imaging compared to both thyroid and PA (124). However, the enhancement pattern alone is not sufficient for accurate diagnosis. Bahl et al. discuss three distinct patterns of PA enhancement defined in comparison to adjacent thyroid gland tissue: (1) Type A–PA significantly darker than thyroid on unenhanced phase, significantly brighter than thyroid on arterial phase, and significantly darker than thyroid on delayed phase; (2) Type B–PA significantly darker than thyroid on unenhanced phase, similar to thyroid on arterial phase, and darker than thyroid on delayed phase; (3) Type C–PA significantly darker than thyroid on unenhanced phase, similar to thyroid on arterial phase, and similar to thyroid on delayed phase (118). In their study, the thyroid had similar Hounsfield units (HU) in the non-contrast and arterial phases, however there were significant differences in the thyroid HU in the delayed phase images between groups A and B *versus* group C, which was not explained. Moreover, only 20% of PA in the series including 94 patients and 110 lesions were brighter than thyroid on the arterial phase, which calls into question the utility of the thyroid as a relevant comparison for arterial hyperenhancement in PA in this study. In another 22% of cases, the pattern was darker than the thyroid in the arterial phase and indistinguishable in the delayed phase, similar to the lymph node enhancement pattern. There was no reported relation between the kinetic pattern relative to the thyroid and the lesion size, weight, or ectopic location. In contrast, Lee et al. described differences in enhancement patterns related to tumor volume with PA>=1cm having greater arterial enhancement and venous washout compared to PA<=1cm which demonstrated more progressive enhancement from arterial to venous phases (121).

Multiple groups attempted to optimize the number of CT phases and accuracy of the test. Hunter et al. found that 3-phases resulted in increased detection accuracy (sensitivity 98%, specificity 97%) compared to arterial only (sensitivity 52%, specificity 75%) in 120 lesions. Contrarily, Raghavan et al. suggested that arterial only (without even an unenhanced phase) was not significantly different from the combination of 2, 3, or 4 phase imaging in a smaller cohort of 29 patients at around 91% accuracy (125). To address this conundrum, a large meta-analysis of 2563 patients found that more post contrast phases of 4DCT in addition to the unenhanced phase resulted in detection improvements concluding that unenhanced plus two post contrast phases was optimal (single contrast: 71%; two-contrast phases: 76%; three-contrast phases: 80%) (126). Ongoing controversy remains regarding the optimal number of phases however, particularly due to the high radiation dose of 4DCT (127). One might consider eliminating the unenhanced phase, however we must remember this phase provides the greatest differences in HU between the thyroid and the PA (118). Dual-energy CT has been suggested capable of reducing dose by 50% (128). Although there is limited evidence regarding its utility in parathyroid disease, dual energy CT could decrease radiation exposure through incorporation of a virtual non-contrast phase (129).

**Figure 9:**
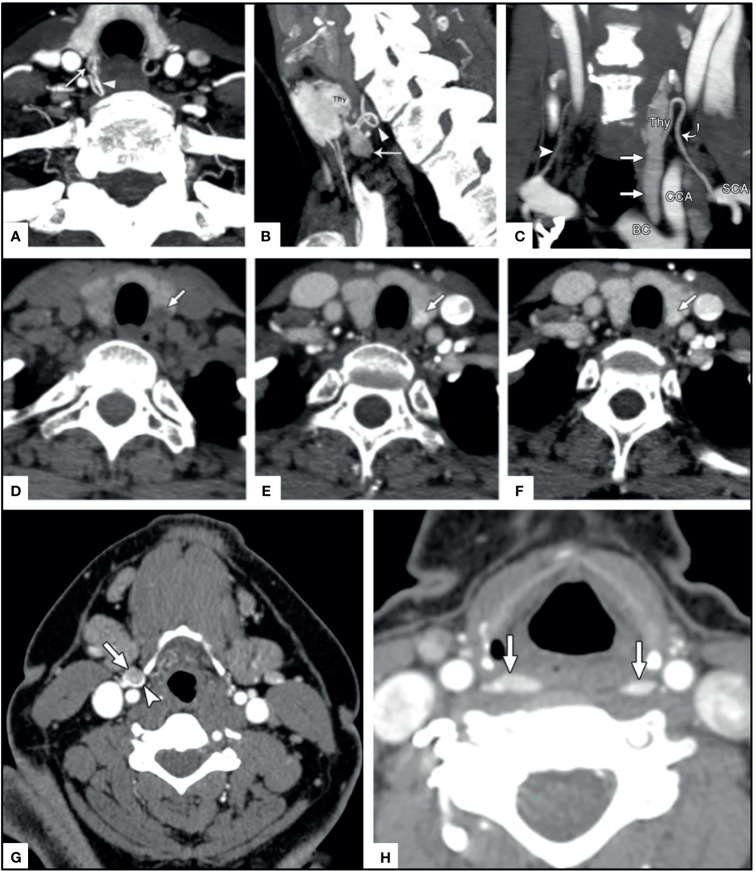
4DCT imaging of parathyroid adenomas. Upper panels **(A)** arrow head on axial image, **(B)** arrow head on sagittal image, and **(C)** curved arrow on coronal images depict the polar vessel sign in PA (straight arrows) with arterially enhancing prominent feeding vessel. Middle panels depict a thyroid nodule (arrow) mimicking a PA on **(D)** non-contrast axial, **(E)** arterial axial, and **(F)** venous axial imaging. Lower panels depict ectopic PA in unusual locations near the carotid sheath (arrow and arrowhead) similar to paraganglioma **(G)** and in the retropharyngeal region (arrows) **(G)** where they could easily be confused for lymphadenopathy. Reproduced with permission from: **(A–C)** *Bahl M, Muzaffar M, Vij G, Sosa JA, Choudhury KR, Hoang JK. Prevalence of the polar vessel sign in parathyroid adenomas on the arterial phase of 4D CT. AJNR Am J Neuroradiol. 2014 Mar;35(3):578-81. doi: 10.3174/ajnr.A3715. Epub 2013 Aug 14. PMID: 23945223; PMCID: PMC7964736.* **(D–H)** *Hoang JK, Sung WK, Bahl M, Phillips CD. How to perform parathyroid 4D CT: tips and traps for technique and interpretation. Radiology. 2014 Jan;270(1):15-24. doi: 10.1148/radiol.13122661. PMID: 24354373*.

In addition to the kinetic characteristics, the polar vessel sign can be seen on 4DCT in the arterial phase and is the finding of an enlarged feeding vessel of a PA, typically the inferior thyroid artery (130). There is not always a clear fat cleft with examples being subcapsular parathyroid and extracapsular sequestration (131) [**Figure 9** (130, 131)].

In a large systematic review of patients with non-familial primary HP having surgery following CT localization, the overall pooled sensitivity was 73% (95% CI: 69-78%) for localization of the correct quadrant and 81% (95% CI: 75-87%) for localization to the correct laterality (126).

Practical advantages of 4DCT could be in the setting of discordant or inconclusive ^99m^Tc-sestamibi and US examination (as a second line test), after unsuccessful initial surgery, or when there is distorted neck anatomy (132–135). To evaluate the utility of 4DCT as a second line test, Tian et al. included 104 patients with inconclusive first line imaging and demonstrated sensitivity of 73% and specificity of 86% compared to ^99m^Tc-sestamibi sensitivity of 48% and ultrasound sensitivity of 52% (136). To evaluate the performance of 4DCT in the re-operative setting, a prospective study of 45 patients showed sensitivity of 88% compared to 54% with ^99m^Tc-sestamibi (135).

Practical limitations of 4DCT include detection of multigland disease (MGD) and ectopic lesions outside of the field of view.

4DCT can be combined with emerging PET/CT agents to achieve even greater diagnostic accuracy. ^18^F-Fluorocholine (FCH) PET/4DCT in 44 patients was found superior ^18^F-FCH PET/CT alone or 4DCT alone achieving 100% sensitivity in 31 of 31 operated patients, which could be considered in patients with primary HP and negative or inconclusive first line imaging (137).

#### Dynamic Enhanced MRI (4DMRI)

MRI has gained attention in localization of the etiology of primary HP due to its ability to perform reliable cross-sectional imaging of the neck and mediastinum without ionizing radiation and with high soft tissue contrast resolution.

Initial evaluation of MRI performance in 2000 by Hanninen et al. found 82% sensitivity for localization of abnormal parathyroid glands or ectopic glands in the mediastinum and submandibular region (138). However, in 2003 Wakamatsu et al. found lower sensitivity of MRI in detection of PA at 43% when using a 0.5 Tesla magnet in conventional approaches (139). Initial challenges in MRI evaluation of the parathyroid, such as motion artifacts and suboptimal fat saturation have been addressed through newer MRI technology and techniques, such as newer time resolved imaging and chemical shift fat saturation imaging sequences.

**Figure 10:**
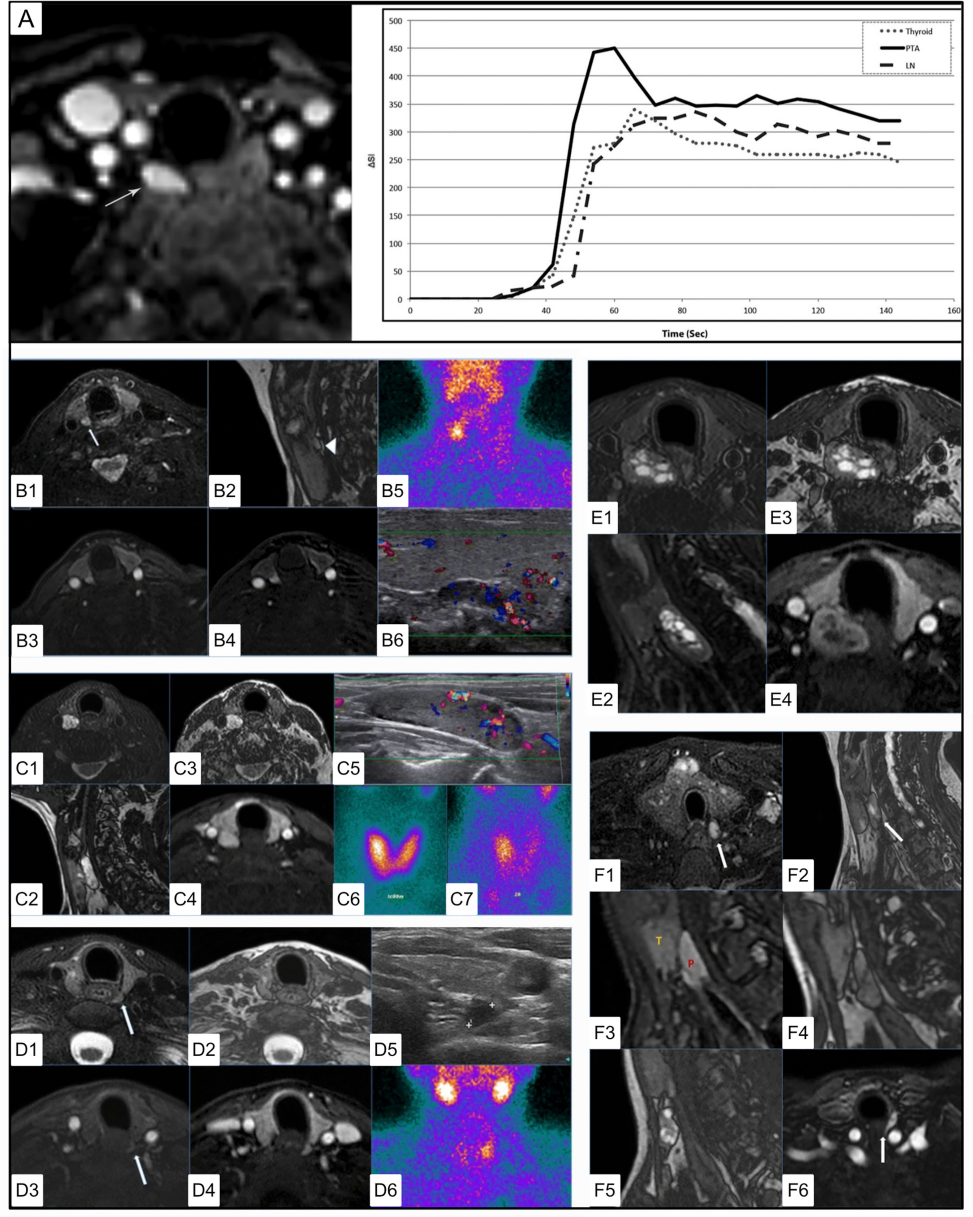
4DMRI imaging of parathyroid adenomas. **(A)** Axial arterial phase post contrast fat saturation T1 MRI of PA at the tracheoesophageal groove (white arrow) in a 47 yo F with primary hyperparathyroidism (PTH - 164 pg/mL, Ca2+ = 10.8). Graph depicts contrast-time curve analysis from ROI placed over the PA (solid), a lymph node (dashed), and the thyroid (dotted), showing relative faster time to peak (TTP), increased wash in and increased wash out values from the PA. **(B)** 4DMRI of PA posterior to the right thyroid mildly hyperintense relative to the thyroid on axial T2 fat saturation [**(B1)**, white arrow] and separated by cleavage plane on sagittal out of phase imaging [**(B2**, white arrow] images with avid arterial enhancement greater than thyroid on T1 post contrast **(B3)** and T1 post contrast subtraction imaging **(B4)** and comparative coronal ^99m^Tc-sestamibi **(B5)** and ultrasound imaging **(B6)**. **(C)** 4DMRI of PA posterior to the right thyroid gland with marked relative T2 fat saturation hyperintensity **(C1)**, oblong appearance without reliable cleavage plane on out of phase imaging in sagittal **(C2)** or axial **(C3)** planes, and arterial enhancement similar to the thyroid gland **(C4)** and associated ultrasound **(C5)** and early **(C6)** and delayed **(C7)** phase ^99m^Tc-sestamibi. **(D)** Dynamic MRI of PA posterior to the left thyroid slightly hyperintense similar to thyroid on axial T2 **(D1)**, with thin cleavage plane on axial out of phase **(D2)**, with mild enhancement on early axial T1 post-contrast subtraction **(D3)** and similar to the thyroid gland on delayed axial T1 post-contrast subtraction **(D4)** and comparative ultrasound **(D5)** and coronal ^99m^Tc-sestamibi **(D6)**. **(E)** 4DMRI of PA posterior to the right thyroid with mixed cystic and solid components causing mass effect on the esophagus displacing it to the left on axial **(E1)** and oblong appearance on sagittal **(E2)** T2 fat saturation, with cleavage plane on axial out of phase **(E3)**, and partial enhancement similar to the thyroid gland on T1 post-contrast fat saturation **(E4)**. **(F)** Summary of MRI features of PA including T2 fat saturation hyperintensity **(F1)**, oblong appearance **(F2)**, cleavage plane between the thyroid and PA **(F3)**, which can be emphasized by india ink artifact on out of phase imaging **(F4)**, ‘marbled’ appearance **(F5)**, fast and strong enhancement on T1 post-contrast **(F6)**. Reproduced with permission from: **(A)** *Nael K, Hur J, Bauer A, Khan R, Sepandari A, Inampudi R, et al. Dynamic 4D MRI for Characterization of Parathyroid Adenomas: Multiparametric Analysis. AJNR Am J Neuroradiol. 2015;36: 2147–2152. doi:10.3174/ajnr.A4425* **(B–F)** *Sacconi B, Argirò R, Diacinti D, et al. MR appearance of parathyroid adenomas at 3 T in patients with primary hyperparathyroidism: what radiologists need to know for pre-operative localization. European Radiology. 2016 Mar;26(3):664-673. DOI: 10.1007/s00330-015-3854-5*.

​​In a study that compared MRI features with findings on ultrasound or ^99m^Tc-sestamibi, five correlative MRI features were identified associated with PA (141) [**Figure 10** (140, 141)]. These included (1) elongated morphology (ratio of longest to shortest diameter >=2.0) with either (2) homogeneous or (3) ‘marbled’ T2 hyperintensity appearance. Out of phase imaging often revealed (4) a fluid fat interface representing the facial plane between the thyroid gland and the PA (although this would not be expected to be present in the case of an intrathyroidal parathyroid adenoma). Dynamic MRI (4DMRI) enhancement characteristics revealed (5) rapid enhancement in post-contrast T1 images.

More recently, Argiro et al. and Becker et al. found excellent sensitivity (up to 97.8%) and specificity (up to 97.5%) for MRI in the detection of PA, which could be due to further optimization of equipment, such as the use of a 3 Tesla magnet and more modern imaging protocols (142, 143). Importantly, Argiro also found good performance in MGD with 8/8 enlarged glands detected and 6/7 ectopic parathyroid glands detected in their series (142). Merchavy et al. found 100% sensitivity for PA in a series of 11 patients using 4DMRI (144).

Anecdotal evidence suggested that MR spectroscopy using the proton high-resolution magic angle spinning with the Algorithm to Determine Expected Metabolite Level Alterations (ADEMA) approach could be used to measure differences in metabolites in primary HP. Using this technique, one group reported increased levels of choline, glycerophosphocholine, phosphorylcholine, glucose, lactate, succinate, glutamine, and ascorbate in single gland disease, however this was not observed in MGD (145). The fact that choline and choline derivatives were selected for MRI spectroscopic analysis supports the use of these agents in molecular imaging techniques, such as choline PET discussed in detail later.

In the standard of care today, MRI is primarily used as a second line modality for problem solving, however 4DMRI could be a modality of choice for localization of PA in patients with MGD or in similar utility as 4DCT while avoiding radiation dose.

In the future, ^18^F-fluorocholine (FCH) PET could augment dynamic structural imaging. Huber et al. found that ^18^F-FCH PET/CT or PET/MRI was able to achieve 96.2% accuracy in detection of PA in 25/26 patients (146). One study found that ^18^F-FCH PET/MRI was even capable of detecting cystic adenomas with comparably lower tracer uptake (147).

#### Angiography

Selective catheterization of the parathyroid drainage pathways allows performance of selective venous sampling (SVS) for localization of PTH secretion (148). This invasive technique was originally developed for patients with recurrent or persistent primary HP following surgery. For successful application of this technique, one must know the thyroid vein anatomy to choose sampling points in the internal jugular and brachiocephalic veins to access the thyroid veins and venous anastomoses. This approach is however limited for assessment of PTH from ectopic PA in the neck or chest due to differences in venous drainage.

**Figure 11:**
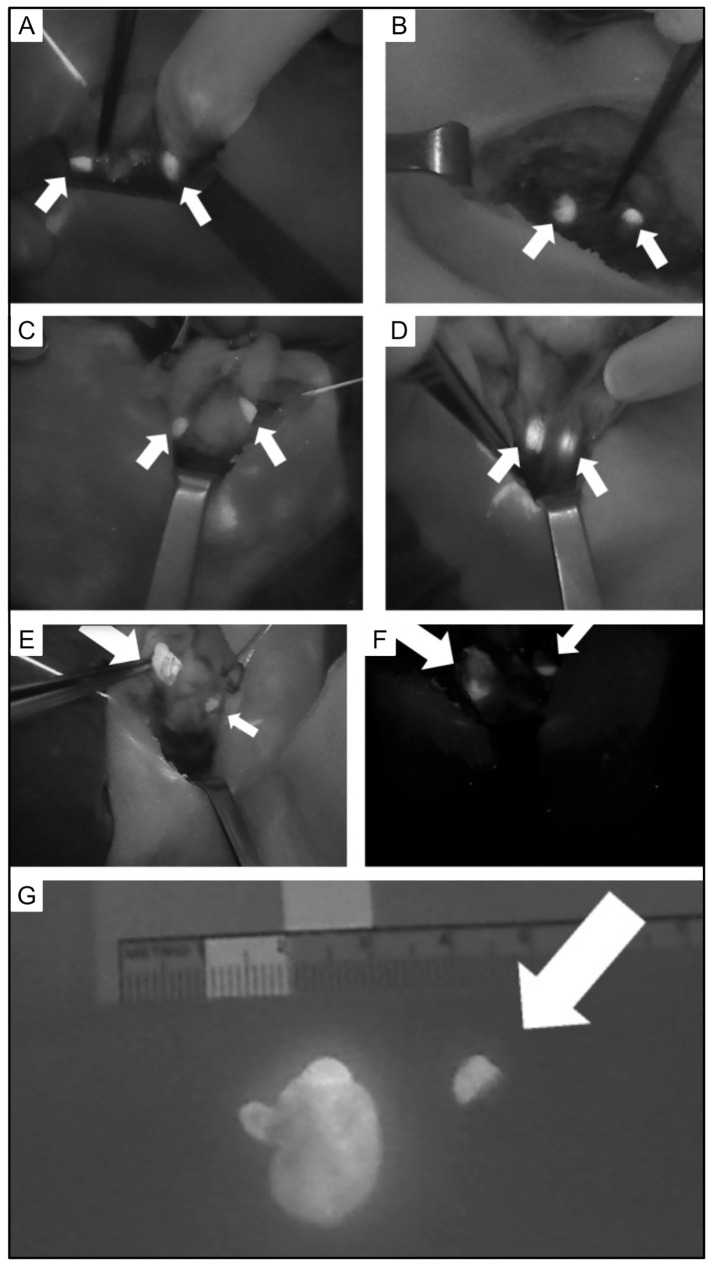
Intraoperative views of parathyroid glands (PG) visualized using autofluorescence (small arrows). **(A)** Two PG following left thyroid lobectomy. **(B)** Two PG following right thyroid lobectomy. **(C)** Two PG following superior pole dissection with medialization of left thyroid. **(D)** Two PG after superior pole dissection with medialization of the right thyroid. **(E)** Superior right PA and **(F)** inferior left PA with heterogeneous fluorescence pattern and enlargement (large arrow) compared to normal PG (small arrow). **(G)** Excised parathyroid adenoma with heterogeneous fluorescence pattern and enlargement (left) compared to normal PG (right, arrow). Reproduced with permission from: *Demarchi, M. S., Karenovics, W., Bedat, B., & Triponez, F. (2020). Intraoperative Autofluorescence and Indocyanine Green Angiography for the Detection and Preservation of Parathyroid Glands. Journal of Clinical Medicine Research, 9(3). https://doi.org/10.3390/jcm9030830*.

A randomized controlled clinical trial evaluated the use of intraoperative Indocyanine green (ICG) parathyroid gland angiography (149). The results suggested that ICG reliably illustrated vascularization of the parathyroid glands. The authors suggested this decreased the need for postoperative measurement of calcium and PTH and that patients with at least one well perfused parathyroid gland did not require calcium supplementation. A recent literature review also suggested this method was effective in reducing postoperative hypoparathyroidism (150) [**Figure 11** (150)].

#### Gamma-Probe Guided Surgery

Intraoperative localization of parathyroid tissue using ^99m^Tc-sestamibi has been explored and is also termed ‘radioguided surgery’ or ‘minimally invasive radioguided parathyroidectomy’. Buicko et al. found that the gamma probe was a useful tool to complement a localization study, particularly in patients with multiple ectopic adenomas or with history of prior parathyroid surgery (151). In a large study of 769 patients with primary HP undergoing radioguided parathyroidectomy using a handheld gamma probe, Chen et al. reported that radioguided techniques were equally effective between patients with negative and positive imaging exams and that the gamma probe allowed detection of all abnormal parathyroid glands, including those that were ectopic (152). The authors suggested a potential benefit in patients with negative preoperative sestamibi scans. Lim et al. recently suggested fewer false positives with intraoperative guidance by a radionuclide probe and reduced operative failure compared to intraoperative parathyroid hormone (IOPTH) measurement in a retrospective cohort of 298 patients (153).

### Comparative Effectiveness

#### US *Versus* ^99m^Tc-Sestamibi

Ultrasound and ^99m^Tc-sestamibi scintigraphy are complementary as the first line of imaging.

There are a couple of controversial studies claiming the sufficiency of ultrasound prior to surgery with the conclusion that it is unnecessary to perform both ultrasound and ^99m^Tc-sestamibi since the latter did not significantly increase diagnostic accuracy (116). One limitation of this study was that it compared the use of planar scintigraphy alone, whereas most nuclear medicine departments today routinely perform SPECT in assessment of primary HP as the standard of care. The other consideration is the design; this study had a single operator during 16 years of the study, meaning it is more representative of the accuracy of that single sonographer rather than the accuracy of ultrasonography, which is known to be subject to high inter-operator variability (68, 154). Apparently, the claims of 90% sensitivity of ultrasonography reported in the literature are highly unlikely, even if by consideration of the high frequency of ectopic parathyroid adenoma alone (155).

#### ^99m^Tc-Sestamibi *Versus* 4DCT: First-Line *Versus* Second Line

Cost and radiation exposure are concerns of any multiphasic CT. 4DCT cost was reported at $1296 compared to $1112 for ^99m^Tc-sestamibi scintigraphy, which can vary more widely based on the radiotracer costs among other factors (156).

Radiation dose of 4DCT is reported to range from 5.56-28 mSv (median 9.3 mSv), typically more than ^99m^Tc-sestamibi planar and SPECT scintigraphy at 3.33-5.6 mSv, but similar to or less than ^99m^Tc-sestamibi SPECT/CT, which is reportedly 12.4 mSv (134, 157), depending on the dose of ^99m^Tc-sestamibi and CT parameters used. We remind the reader however that the radiation dose to the thyroid is reported to be 57 times higher with 4DCT compared to scintigraphy (120), although this will vary by equipment and specific institutional protocols.

Comparative effectiveness of 4DCT relative to other parathyroid disease imaging techniques is an area of considerable debate with various protocols explored in the literature (158–161). Investigations on comparative effectiveness for detection of parathyroid adenoma must be carefully and critically evaluated for experimental design in order to better understand the meaning of the results.

To objectively compare the accuracy of ultrasound, sestamibi, and 4DCT, Kedarisetty et al. evaluated 58 patients and found the accuracy of 4DCT was not significantly different compared to ^99m^Tc-sestamibi SPECT/CT (162). Although 4DCT did find 3 true positives that were initially false negatives, this was not sufficient to raise the sensitivity, specificity, or accuracy enough to be statistically significant. They found 4DCT was useful for localizing ectopic glands occurring in the mediastinum, thymus, tracheoesophageal groove, and retrosternal space, but was less helpful in the setting of multigland disease (MGD). These findings were consistent with other reports that 4DCT can be less accurate in diagnosis of smaller lesions and cases with multiple culprit lesions. They encouraged the main utility of 4DCT is in the setting of negative ^99m^Tc-sestamibi SPECT/CT. In 28 patients where 4DCT was performed out of 1485 total adult patients in their retrospective cohort study, Broome et al. suggested 4DCT performed better than 99Tc-sestamibi SPECT in single adenomas and better than other modalities investigated for MGD and double parathyroid adenoma (163). These findings were however underpowered and compared 4DCT to SPECT rather than SPECT/CT.

The EANM 2021 practice guidelines (164) suggest 4DCT has similar diagnostic performance compared to ^99m^Tc-sestamibi SPECT based on meta-analysis (165), however acknowledges protocols vary among institutions and the referenced meta-analysis suggested cervical ultrasound and ^99m^Tc-sestamibi SPECT or SPECT/CT as first line approaches.

### Future Directions

#### Positron Emission Tomography

PET imaging has improved sensitivity and spatial resolution over SPECT imaging. PET provides better accuracy and clearer images with faster acquisition compared to SPECT (166). Several molecular tracers are currently being evaluated for PET imaging of the parathyroid.

#### ^18^F-Fluorocholine (FCH) PET/CT

Although earlier work in PET imaging for patients with primary HP suggested ^11^C-methionine as a promising agent, more recent studies have focused on choline due to its increased accuracy (167). ^11^C-choline and ^18^F-fluorocholine (FCH) have been reported useful in imaging parathyroid hyperplasia and adenomas. ^11^C requires an on-site cyclotron having only a 20 min half-life. Therefore ^18^F-FCH is a more practical PET tracer for potential commercial use.

**Figure 12:**
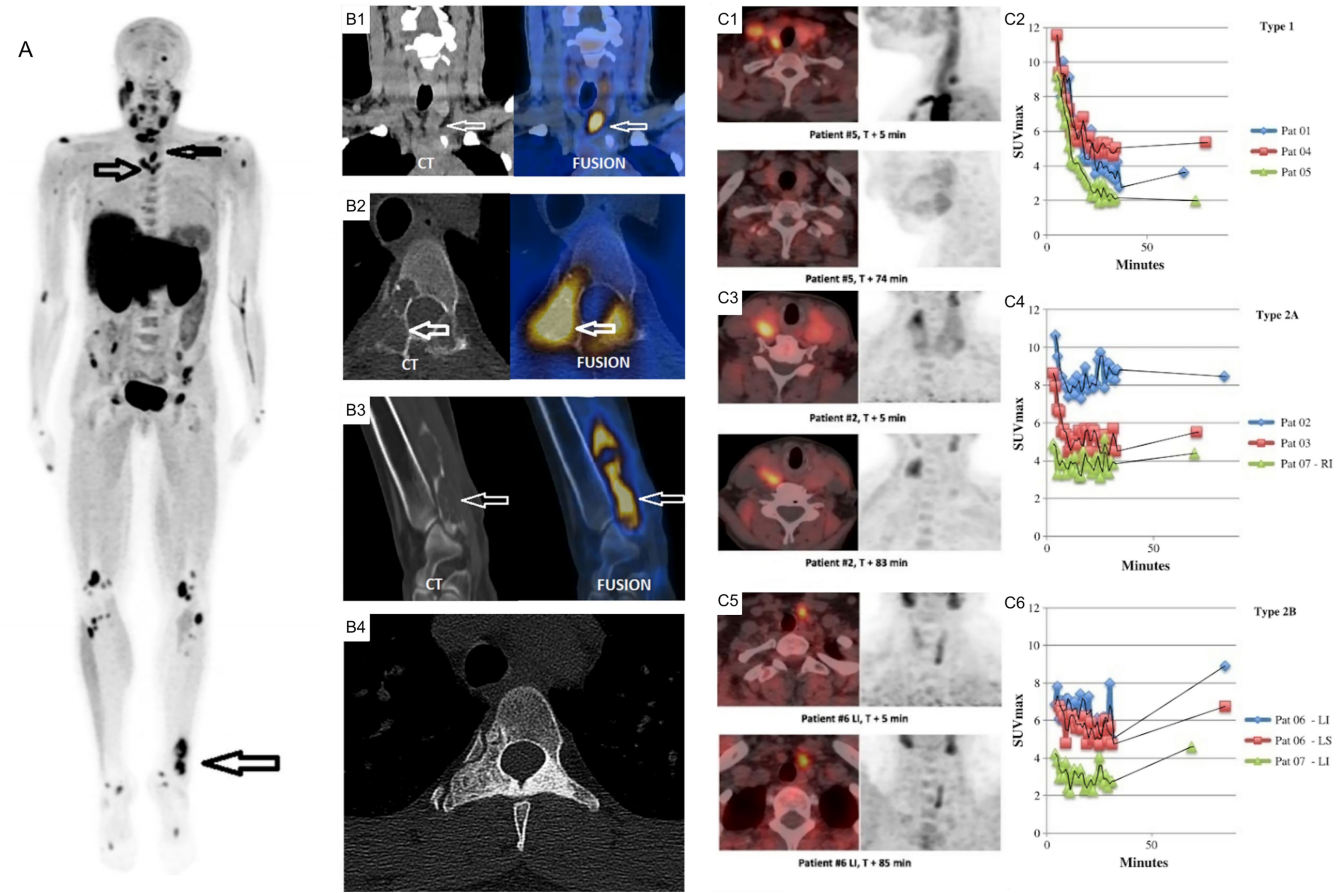
**(A)** 18F-FCH PET/CT whole body imaging in a patient with severe hyperparathyroidism depicting multiple brown tumors on maximum intensity projection (MIP) images (arrows), coronal CT (left) and fusion PET/CT (right) images depicting parathyroid adenoma **(B1)**, axial **(B2)** and oblique **(B3)** CT (left) and fusion PET/CT (right) images depicting brown tumor (arrows), axial CT depicting sclerotic healed brown tumor **(B4)** following cure of hyperparathyroidism. **(C)** Comparison of acquisition protocols for 18F-FCH PET/CT showing cases where an earlier <10 min time point had higher uptake compared to a delayed >60min time point **(C1, C2)**, cases where earlier and delayed time points where similar **(C3, C4)**, and cases where the delayed time point had increased uptake **(C5, C6)**. 18F-FCH, 18F-fluorocholine. Reproduced with permission from: **(A, B)** *Zhang-Yin J, Gaujoux S, Delbot T, GauthéM, Talbot JN. 18F-Fluorocholine PET/CT Imaging of Brown Tumors in a Patient With Severe Primary Hyperparathyroidism. Clin Nucl Med. 2019 Dec;44(12):971-974. doi: 0.1097/RLU.0000000000002814. PMID: 31652163*. **(C)** *Morland D, Richard C, Godard F, Deguelte S, Delemer B. Temporal Uptake Patterns of 18F-Fluorocholine Among Hyperfunctioning Parathyroid Glands. Clin Nucl Med. 2018 Jul;43(7):504-505. doi: 10.1097/RLU.0000000000002132. PMID: 29762240*.

The major pitfall of choline is that it traces general neoplastic processes and is not a targeted biomarker of parathyroid disease. Uptake of choline in neoplastic cells is felt to be due to the increased demand for phospholipid synthesis in cells with a high proliferative rate (168). In benign parathyroid adenomas, it has been postulated that increased lipid-dependent choline kinase activity due to PTH hypersecretion may account for increased choline uptake (169). ^18^F-FCH PET is also present in brown tumors [**Figure 12** (170, 171)].

##### Overall Performance

In their recent systematic review of the literature, Treglia et al. reported a meta-analysis including 14 studies containing a total of 517 patients. They reported that PET imaging with choline for detecting hyperfunctioning parathyroid glands in patients with hyperparathyroidism on a per patient basis had a sensitivity of 95% (95% CI: 92-97%), positive predictive value 97% (95% CI: 95-98%), and detection rate 91% (95% CI: 87-94%). Per lesion analysis yielded similar results with 92% sensitivity and positive predictive value (PPV) (172). Another recent systematic review yielded PPV 97% and 92% for per patient and per lesion analysis respectively (173).

Evangelista and colleagues compared ^18^F-FCH PET imaging in conjunction with CT or MRI to conventional structural and functional imaging in perhaps the largest reported cohort of patients in the literature today in a systematic review (174). In their study including 23 articles and 1112 patients, they found ^18^F-FCH PET to be more accurate compared with ultrasonography, ^99m^Tc-sestamibi SPECT, or MRI alone. This article did not differentiate between imaging protocols, which varied among the studies. There is no technical limitation precluding the use of 4D conventional imaging approaches in CT and MRI in conjunction with ^18^F-FCH PET/CT or ^18^F-FCH PET/MRI acquisition protocols simultaneously.

##### Optimal Timing

Assessment of ^18^F-FCH PET protocol parameters recently suggested that after injection of 2.5 MBq/kg imaging 60 minutes after 2 minute injection resulted in the best sensitivity, specificity, and target to background ratio (TBR) compared to 5, 10, 15, and 20 minutes post-injection (175). Another highly cited article in a smaller series of patients suggested that ^18^F-FCH PET TBR could at times be limited in efficacy due to rapid washout after 5-9 minutes in some instances (176).

##### Potential Pitfall

Differentiated thyroid cancer (DTC) has been reported to demonstrate ^18^F-FCH PET uptake, particularly in less differentiated subtypes and should be recognized as a potential mimicker of hyperplastic parathyroid tissue (177, 178). When ^18^F-FCH PET uptake is present in the thyroid, DTC should be suspected as it is more common than intrathyroidal parathyroid tissue (166). ^18^F-FCH PET has been suggested to demonstrate uptake in inflammatory lymph nodes, however more evidence is needed to determine whether or not this could be a point of confusion in the setting of parathyroid imaging (179, 180).

##### Synergism: ^18^F-FCH PET/4DCT

It has been suggested hybrid PET/4DCT could also be helpful for improved structural characterization in synergy with ^18^F-FCH PET, even increasing sensitivity to 100% in one study (137, 160, 181).

#### ^18^F-Fluorocholine (FCH) PET/MRI

**Figure 13:**
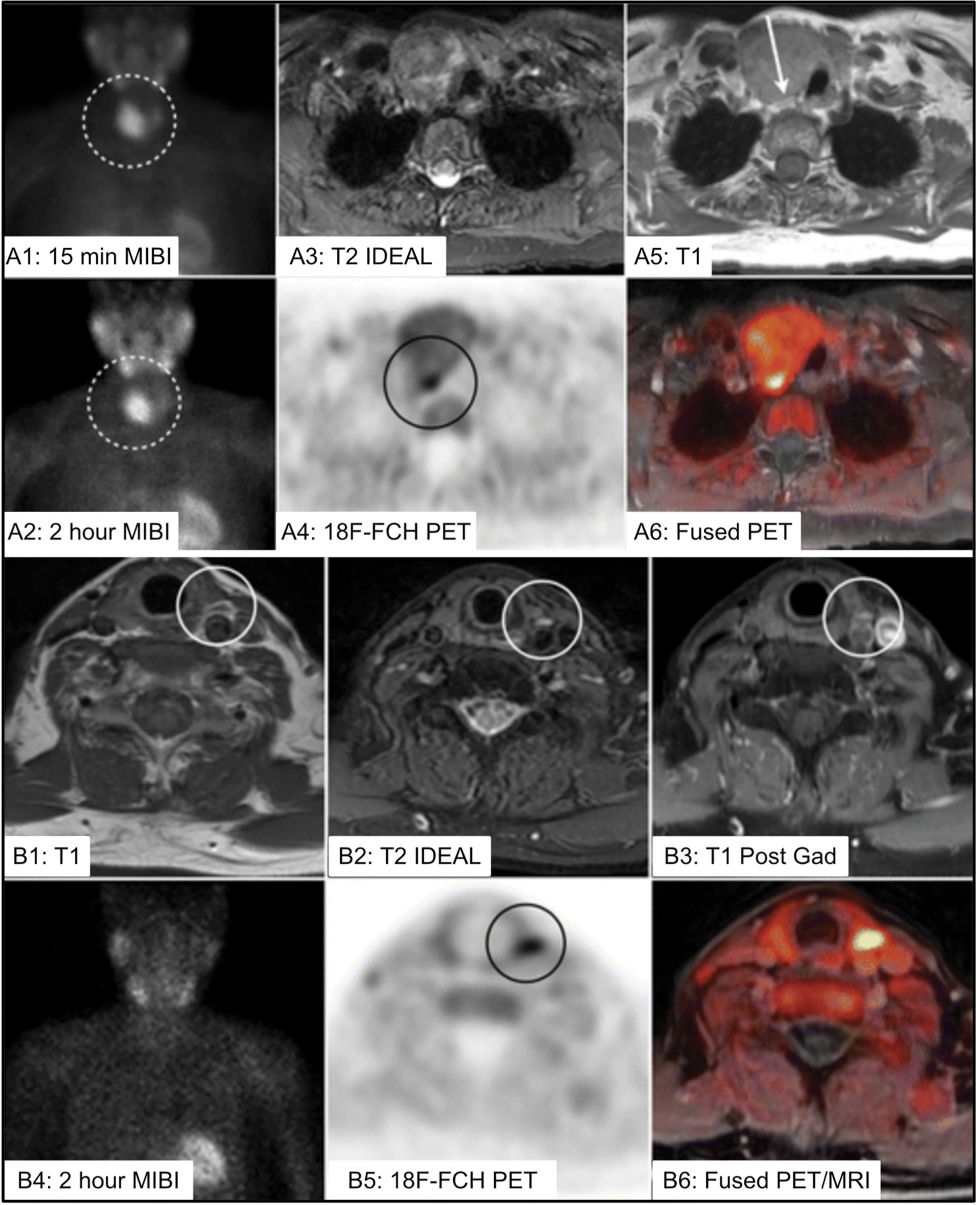
**(A)** 73 yo F with multinodular goiter and 5cm dominant nodule [**(A1, A2)**, dotted circle] with uptake on 99mTc-sestamibi (MIBI) limiting ability to localize the PA. 18F-FCH PET/MR imaging identified PA posterior to the dominant nodule **(A3–A6)** [**(A4)**, black circle, **(A5)**, white arrow]. **(B)** 75 yo F with left PA identified on 4DMRI [**(B1–B3)**, white circle], with inconclusive MIBI scan **(B2)**, and correlative increased 18F-FCH uptake within the PA on PET/MRI imaging **(B5, B6)** [**(B5)**, black circle]. IDEAL, Iterative decomposition of water and fat with echo asymmetry and least-squares estimation; 18F-FCH, 18F-fluorocholine; MIBI, 99mTc-sestamibi; PA, parathyroid adenoma. Reproduced with permission from: *Kluijfhout WP, Pasternak JD, Gosnell JE, Shen WT, Duh QY, Vriens MR, de Keizer B, Hope TA, Glastonbury CM, Pampaloni MH, Suh I. 18F Fluorocholine PET/MR Imaging in Patients with Primary Hyperparathyroidism and Inconclusive Conventional Imaging: A Prospective Pilot Study. Radiology. 2017 Aug;284(2):460-467. doi: 10.1148/radiol.2016160768. Epub 2017 Jan 25. PMID: 28121522*.

^18^F-FCH PET/MRI may provide improved structural characterization of parathyroid lesions, particularly in pediatric patients and in more challenging/subtle cases such as secondary hyperparathyroidism (182–184) [**Figure 13** (184)]. Two recent articles supported improved accuracy of ^18^F-FCH PET/MRI in detection of PA in a cohort of 98 patients and an expanded cohort of 101 patients (180, 185). The authors reported that ^18^F-FCH PET/MRI was more successful in guiding curative surgery (83%) compared to ultrasound (35%) and ^99m^Tc-sestamibi (24%).

#### Dynamic PET

Use of dual time point PET and dynamic PET have also been initially explored (186–188). Total Body PET has been reported to be a promising approach to dramatically improve the temporal resolution of dynamic PET imaging (189, 190).

#### Flurpiridaz

^18^F-Flurpiridaz is derived from the insecticide pyridaben, an analog of rotenone. ^18^F-Flurpiridaz is a lipophilic antagonist of mitochondrial complex I (NADH:ubiquinone oxidoreductase) competing for the ubiquinone binding site at the inner mitochondrial membrane. As ^18^F-Flurpiridaz’s efflux half-time from the mitochondria is greater than the half-life of the ^18^F isotope any potential imaging impact of redistribution is minimized (166, 191–196). ^18^F-flurpiridaz (^18^F-BMS, ^18^F-BMS-747158-02) is under phase 3 clinical investigation for myocardial perfusion imaging. ^18^F-flurpiridaz has shown promise in imaging mitochondria in other organs, such as liver (197), and has the potential to become an important parathyroid imaging agent (166). The agent is promising because it targets mitochondria similar to ^99m^Tc-sestamibi, the current molecular imaging standard of care, due to the high mitochondria content of the parathyroid. In a study of 132 patients comparing ^18^F-flurpiridaz PET to ^99m^Tc-sestamibi SPECT in myocardial perfusion, ^18^F-flurpiridaz PET was consistently rated to provide improved image quality and approximately 3-fold diagnostic certainty (198). It is hoped that the same could hold true in parathyroid imaging.

^18^F-(4-Fluorophenyl)triphenylphosphonium is another agent originally investigated in nuclear cardiology that could be explored. It traces mitochondria on a similar principle to sestamibi as a lipophilic cation that is trapped due to the high mitochondria membrane potential (199).

### Summary Approach to Hyperparathyroidism

​​The approach to hyperparathyroidism begins with initial clinical and laboratory investigation to establish the diagnosis of primary HP. From there, non-invasive imaging provides a cost effective and safe method of localizing the source of PTH, which in turn contributes towards the goal of successful initial surgery.

Parathyroid imaging is a relatively infrequent procedure since the disease affects only 0.7% of the general population (200). Because of this low incidence, there are few high-volume imaging centers in the United States. Approximately 35% of the parathyroid surgeries are performed in the low-volume centers (201). The wide range of reported sensitivity for ^99m^Tc-sestamibi SPECT/CT, from 28% to 98%, indicates variation in local expertise. High quality molecular imaging might not be available in low volume centers and 4DCT might be the best available option.

Selection of first line imaging is controversial even among high volume centers and depends on institute expertise. In our institute, the standard of care initial approach to localization of PA begins with ultrasonography and dual tracer, dual phase ^99m^Tc-pertechnetate/^99m^Tc-sestamibi with planar scintigraphy and delayed SPECT/CT.

When first line imaging is inconclusive or discordant, dynamic contrast enhancement either through 4DCT or 4DMRI can be a helpful problem-solving tool. The diagnostic approach to localization should aim to focus on case-by-case interpretation with the goal to distinguish any potential PA from the thyroid and lymph nodes on each series possible. There is data to suggest a benefit for 4DCT in the setting of failed prior surgery or distorted neck anatomy. Even though 4DCT has been suggested to have similar diagnostic accuracy to ^99m^Tc-sestamibi SPECT as a first line modality, the radiation exposure to the thyroid is much higher.

#### Outstanding Issues

Several issues remain areas of investigation that deserve more exploration in future research.

Multigland disease (MGD) includes multiple PA or hyperplasia. MGD is challenging and can be susceptible to satisfaction of search. MGD occurs in 15-20% of cases and should not be thought of as rare. The trend toward minimally invasive parathyroidectomy can contribute to missed MGD. In cases of suspected MGD not identified by standard imaging techniques, open neck exploration is and will be the standard of care.

Ectopic glands or recurrence in unusual regions remains a clinical challenge. In the pericardial region, ectopic PA can occur, but are difficult to localize due to cardiac activity for instance. Although issues such as these are rare, they do occur and continue to pose a diagnostic challenge.

The Achilles heel of current parathyroid imaging techniques is that none of the current methods of diagnostic imaging for PA are precisely targeted to disease biology. On dynamic imaging, wash in and wash out characteristics vary. ^99m^Tc-sestamibi was not designed for PA imaging and thyroid nodules or malignancy can be confounding.

A targeted agent specific for a parathyroid tissue biomarker is awaited.

## Author Contributions

All authors contributed to the conception and design of the review. MM and BS wrote the first draft of the manuscript and contributed equally. MM, BS, and CC composed the figures. MM, BS, CM, CC, EJ, AM and MA wrote, reviewed for accuracy, and revised sections of the manuscript. All authors contributed to manuscript revision, read, and approved the submitted version.

## Funding

This research was supported in part by the Intramural Research Program of the NIH, Clinical Center. The opinions expressed in this publication are the author’s own and do not reflect the view of the National Institutes of Health, the Department of Health and Human Services, or the United States government.

## Conflict of Interest

The authors declare that the research was conducted in the absence of any commercial or financial relationships that could be construed as a potential conflict of interest.

## Publisher’s Note

All claims expressed in this article are solely those of the authors and do not necessarily represent those of their affiliated organizations, or those of the publisher, the editors and the reviewers. Any product that may be evaluated in this article, or claim that may be made by its manufacturer, is not guaranteed or endorsed by the publisher.

## Footnotes

1 This case presentation was in a ceremony to celebrate Virchow’s 71st birthday.
